# Probiotic supplementation regulated swine growth performance, fecal odor reduction and carcass characteristics by modulating intestinal microbiome

**DOI:** 10.1186/s42523-025-00441-9

**Published:** 2025-07-16

**Authors:** Yung-Tsung Chen, Yu-Ting Sun, Herng-Fu Lee, Yu-Chun Lin, Ming-Ju Chen

**Affiliations:** 1 Department of Food Science, National Taiwan Ocean University, Keelung, Taiwan; 2 Department of Animal Science and Technology, National Taiwan University, Taipei, Taiwan; 3 Taiwan Livestock Research Institute, Ministry of Agriculture, Tainan, Taiwan; 4 Fisheries Research Institute, Ministry of Agriculture, Keelung, Taiwan

**Keywords:** Probiotics, Gut microbiota, Growth performance, Fecal odor, Carcass characteristics

## Abstract

**Background:**

Using probiotics as a substitute for antibiotic growth promoters and reducing odor has received increasing attention in animal science. Despite the extensive investigation into the effects of probiotic administration on swine growth performance and odor reduction by short study durations, the analysis of carcass characteristics and potential mechanistic insights involving gut microbiota and downstream pathways is still few.

**Methods:**

A total of 48 crossbred LYD [(Landrace x Yorkshire) x Duroc] piglets (equal numbers of males and females) were randomly assigned to one of four dietary treatments: control (CON), *Lactobacillus kefiranofaciens* M1 (M1), M1 + *Bacillus amyloliquefaciens* S20 (SA group) and M1 + S20 + *Bacillus subtilis* S14 (SAM group). During the nursery phase (4–8 weeks), pigs were pair-housed and monitored for diarrhea. From 8 to 19 weeks of age, pigs were individually housed and fed grower-finisher diets. Growth performance, blood biochemistry, fecal enzyme activity, and odor-related metabolites were assessed at multiple time points. At market weight (~ 110 kg), six pigs per group were slaughtered for carcass and cecal microbiota analysis.

**Results:**

The results demonstrated that administration of the probiotics led to increased body weight and average daily weight gain, particularly notable during the weaning and finishing periods. Additionally, the SA and SAM groups significantly reduced skatole concentration in feces. Furthermore, probiotic supplementation was associated with increased carcass weight, with the SAM group exhibiting significantly higher tenderloin weight than the CON group. Microbiota analysis revealed taxa exhibiting significant differences in abundance among groups, with corresponding LEfSe findings.

**Conclusion:**

Administering *Bacillus subtilis* S14 and *B. amyloliquefaciens* S20 (SA group) impacted growth performance, reduced fecal odor, and enhanced pig carcass quality. The identified probiotic strains hold promise as feed additives, offering a potential solution to challenges encountered by the swine industry.

**Graphical abstract:**

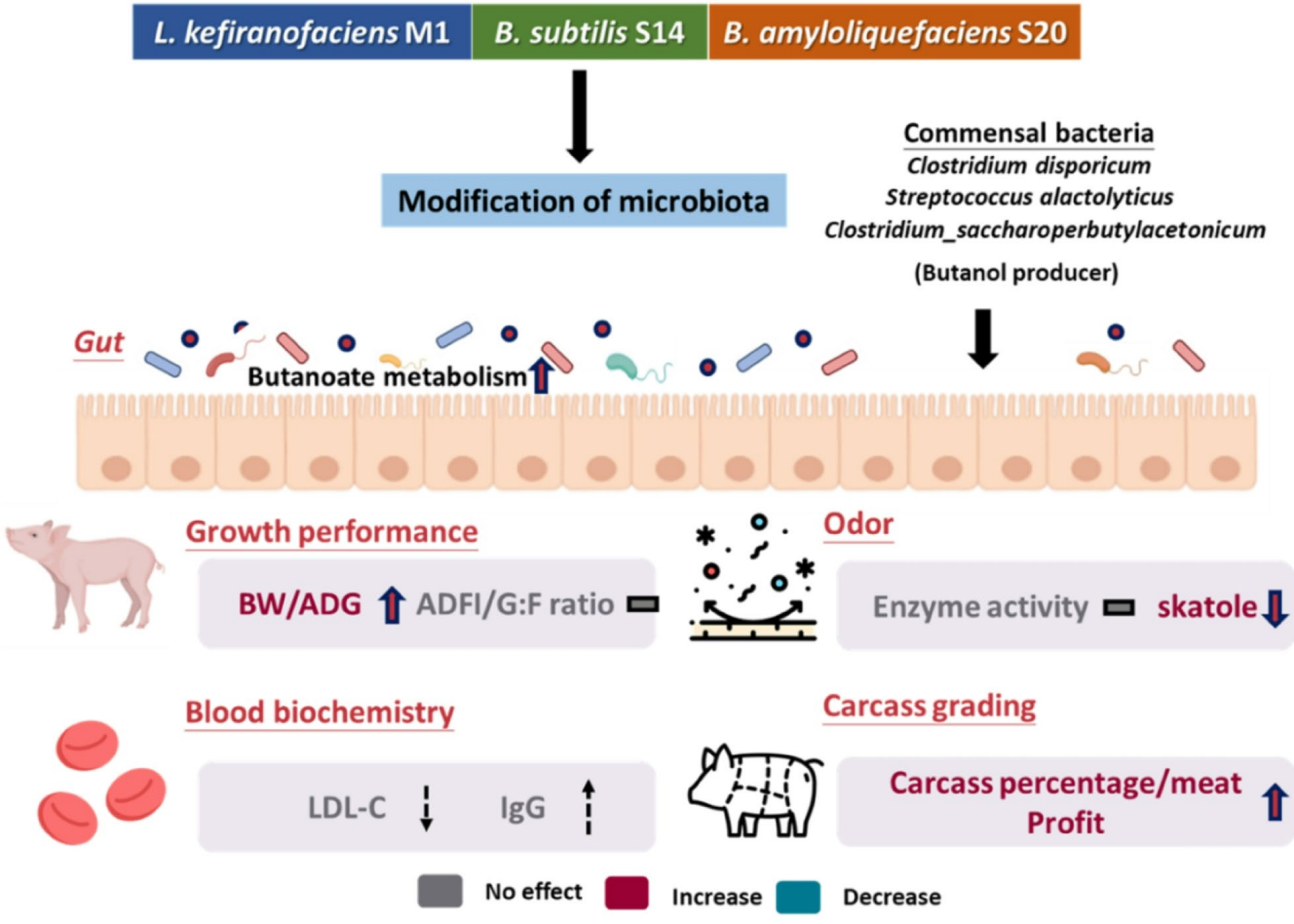

**Supplementary Information:**

The online version contains supplementary material available at 10.1186/s42523-025-00441-9.

## Introduction

The use of antibiotics and the expansion of livestock farming present significant challenges for the pig farming sector. While antibiotics as feed additives can enhance animal health due to their antimicrobial effects and associated benefits, their widespread use has led to the emergence of antibiotic-resistant bacteria [1]. Moreover, the emission and dissemination of odors from animal production facilities, primarily comprising sulfur compounds, phenols, indoles, volatile fatty acids (VFAs), ammonia, and volatile amines [2], are associated with various human health issues. These include respiratory problems such as decreased lung function, bronchitis, sinusitis, nasal inflammation, sore throat, and headaches [3–5].

The gastrointestinal tract harbors a complex ecosystem comprising numerous microorganisms with diverse metabolic capabilities [6]. Interactions among the host, diet, and gut microbiome can impact pig health and the emission of odors, potentially by generating various metabolites with multifaceted functions. Multiple studies conducted in pigs have underscored the significance of the gut microbiome and its metabolites in enhancing growth performance [7], improving feed efficiency [8], reducing stress [9], and offering protection against pathogens [10].

Probiotics represent one of the strategies employed to manipulate the gut microbiome of pigs, with promising outcomes reported in optimizing pig production [11]. Numerous studies demonstrated that incorporating probiotics from diverse genera like *Bacillus* [12], *Saccharomyces* [13], and lactic acid bacteria [14] into animal feed can enhance growth performance and health in pig. Probiotics, particularly strains like *Bacillus* spp., have also garnered attention in research focused on reducing odor emissions from pig manure [15, 16]. These microbial supplements are believed to operate by modulating the intestinal microbial community, which in turn influences diverse metabolic processes such as nutrient absorption, lipid metabolism, and amino acid synthesis [17].

While probiotics have garnered significant attention and utilization within the pig farming industry, existing research predominantly concentrates on specific growth phases. There remains a notable gap in understanding the comprehensive impact of probiotics across the entire pig rearing continuum, spanning from piglets to finishing pigs. Moreover, the intricate functions of probiotics within the intestines have received limited exploration. In our previous study, *Lactobacillus kefiranofaciens* M1 demonstrated a immunoregulatory [18] and bodyweight enhancing effects [19]. Two additional Bacillus species, *Bacillus subtilis* S14 and *B. amyloliquefaciens* S20, have demonstrated potential for reducing fecal odor in vitro. Therefore, we investigated the effects of these three bacterial strains on pig health, growth performance, fecal odor emission, and carcass quality. This study endeavors to elucidate the potential mechanisms and pathways through which probiotics may bolster animal well-being, mitigate pig manure odor, and modulate microbial composition in pig intestines.

## Materials and methods

### Animal housing and experimental design

A total of 48 LYD [(Landrace x Yorkshire) x Duroc] crossbred piglets (24 males and 24 females) with similar body weights at 4 weeks of age were included in the study. During the nursery phase (up to 10 weeks of age), each pen housed a pair of pigs (one barrow and one gilt) fed experimental diets (see Table S1). All diets were formulated based on the nutrient requirements for swine as outlined by the NRC (2012) [20]. The feeding program consisted of four dietary phases: nursery phase (4–10 weeks of age), growth phase (10–17 weeks of age), and finishing phase (17–23 weeks of age). Transitions between dietary phases were determined according to the pigs’ chronological age, in line with the standard swine production protocol and NRC (2012) [20] nutrient requirement guidelines.

The room temperature and humidity of nursery house and growing-finishing house were recorded every ten minutes automatically. A heating lamp was hanged upon each pen of the nursing house to keep warm for the pigs. The average of temperature and humidity of nursery house was 29.1℃ (ranged 27.6℃– 30.3℃) and 70.6% (ranged 65.3– 72.8%), respectively and the average of temperature and humidity of growing-finishing house was 25.6℃ (ranged 15.3℃– 30.2℃) and 74.6% (ranged 59.5– 84.6%, respectively. The light cycle of both houses was controlled by a timer that the light was turned on at 06:00 and turned off at 18:00 automatically every day.

The daily incidence of diarrhea was monitored in the piglets. After the nursery phase, the pigs were moved to individual pens and continued on grower-finisher diets until they reached 23 weeks of age.

Twelve pigs were assigned to each of the four dietary treatments: CON (control, basal diet with no probiotics), M1 (basal diet supplemented with 2 × 10¹⁰ CFU/kg feed of *Lactobacillus kefiranofaciens* M1), SA (basal diet supplemented with 2 × 10¹⁰ CFU/kg feed of *Bacillus subtilis* S14 and *Bacillus amyloliquefaciens* S20), and SAM (basal diet supplemented with 2 × 10¹⁰ CFU/kg feed of *L. kefiranofaciens* M1, *B. subtilis* S14, and *B. amyloliquefaciens* S20). All the pigs were supplied feed and water *ad libitum*.

*L. kefiranofaciens* M1 was isolated from kefir grains, characterized, and preserved in our laboratory, with documented anti-inflammatory and anti-colitis properties [21, 22]. This strain was cultured in MRS agar and harvested during the late exponential phase. *B. subtilis* S14 and *B. amyloliquefaciens* S20 were isolated from pig feces and were previously shown in our in vitro fermentation studies to reduce levels of indole, 3-methylindole, and p-cresol. Both *Bacillus* strains were cultured in Bacillus Medium (Stbio Co., Taipie, Taiwan) and harvested at the late exponential phase. All bacterial cell pellets were collected by centrifugation, washed with sterile phosphate-buffered saline (PBS), and lyophilized to obtain a dry powder.

The rationale for selecting these strains and their combinations was based on previous findings demonstrating their beneficial effects in vitro, and dosage was calculated based on body surface area conversion. The lyophilized probiotic powders were stored at room temperature in airtight, opaque containers until use. Immediately prior to feeding, the required quantity of each probiotic was thoroughly mixed into the basal diet to achieve the targeted viable counts. To ensure viability, the viable cell count in the supplemented feed was periodically verified using plate count methods. All procedures followed standard guidelines for probiotic feed supplementation in animal trials [23].

Pigs were individually weighed at each designated time point following an overnight fasting period, in accordance with standard swine research protocols to ensure measurement accuracy. Body weights were recorded at the start of the experiment and every two weeks thereafter to calculate average daily gain (ADG), average daily feed intake (ADFI), and feed efficiency (G: F ratio). Blood and fecal samples were collected at week 0 (day 0), week 6 (day 42), week 13 (day 91), and week 19 (day 133) of the experimental period for the analysis of blood biochemistry, fecal enzyme activities, and odor-related metabolites. At market weight (approximately 110 kg), six pigs per group were randomly selected from those that had reached the target body weight for slaughter and subsequent carcass and cecal microbiota analysis. The random selection was conducted using a random number generator to minimize selection bias and to ensure that the sampled individuals were representative of their respective groups, as recommended by animal research guidelines [20, 24].

The Institutional Animal Care and Use Committee (IACUC) of the Taiwan Livestock Research Institute, Ministry of Agriculture, Taiwan approved the experiment (Approval number 109 − 54). All animal experimental procedures adhered to the regulations stipulated by the Taiwan Livestock Research Institute, Ministry of Agriculture, Taiwan.

### Diarrhea incidence in piglets

The diarrhea status of each pen was monitored daily during the nursery period and scored on a four-point scale: 0 (normal), 1 (soft feces), 2 (mild diarrhea), and 3 (severe diarrhea), as previously described [25]. For group-level analysis, the incidence of diarrhea was calculated as the percentage of pen-day observations with a score of 1, 2, or 3 relatives to the total number of observations for each group. For example, if 10 out of 42 pen-day observations in a week had scores ≥ 1, the diarrhea frequency for that week would be 23.8%. The percentages reported in Table 1 reflect this calculation method.

**Table 1:** Effect of probiotics treatment on diarrhea score and frequency in piglets

Weeks	Diarrhea frequency (%)			
	CON	M1	SA	SAM
	%	%	%	%
W1	42.86	26.19	38.10	28.57
W2	16.67	16.67	30.95	28.57
W3	23.81	23.81	40.48	30.95
W4	50.00	23.81	45.24	21.43
	Diarrhea score			
W1	0.83 ± 0.31^Aa^	0.50 ± 0.22^Ab^	0.74 ± 0.29^Aab^	0.52 ± 0.31^Ab^
W2	0.34 ± 0.20^Ca^	0.26 ± 0.19^Ba^	0.45 ± 0.33^Ba^	0.30 ± 0.23^Ba^
W3	0.66 ± 0.25^Bb^	0.29 ± 0.27^BCc^	0.86 ± 0.42^Aa^	0.38 ± 0.32^ABc^
W4	0.64 ± 0.20^Bb^	0.39 ± 0.33^ABc^	0.92 ± 0.49^Aa^	0.31 ± 0.30^Bc^

Diarrhea score: 0 = Normal; 1 = Soft feces; 2 = Mild diarrhea; 3 = Severe diarrhea

*N* = 6 (pen numbers)

^A, B,C^ Means within a column that do not share a common superscript are significantly different (*P* < 0.05)

^a, b,c^ Means within a row that do not share a common superscript are significantly different (*P* < 0.05)

### Measurement of growth performance

Pigs were weighed individually at the beginning of the experiment each month without fasting. Daily feeding amounts and leftovers were recorded between 09:00 and 09:30 AM. These values were used to calculate average daily feed intake (ADFI; g/d), average daily gain (ADG; g/d), and gain-to-feed ratio (G: F ratio). For growth performance, we paired the data from the two pigs in each pen during the nursery period and used this paired data as the experimental unit for statistical analysis across all periods: whole, nursery, growing, and finishing.

### Blood and fecal sampling

The fecal and blood sampling methods were described as below. Briefly, approximately one hour after feeding, the pig was restrained using a stainless-steel snare. A sterile cotton swab moistened with sterile saline was gently used to stimulate the pig’s anus. When the pig defecated, a sterile sample bottle was positioned to collect the mid-section of the feces. The bottle was securely capped, and the date and sample number were clearly labeled on the lid.

For blood sampling, on the day of collection and prior to feeding, the pig was securely restrained with a stainless-steel snare. Blood samples were collected from each pig via venipuncture of the anterior vena cava using standard aseptic techniques [26, 27]. For plasma analysis, blood was drawn into EDTA-coated anticoagulant tubes and centrifuged at 1,500 × g for 15 min at 4 °C; the plasma was then aliquoted and stored at − 20 °C until analysis. For serum collection, blood samples were obtained using tubes containing a clot activator, allowed to clot at room temperature for 30 min, and centrifuged under the same conditions; the serum was subsequently separated and stored at − 20 °C. All analyses were performed according to the manufacturers’ protocols and standard laboratory procedures.

### Analysis of blood biochemistry

Whole blood and serum samples (1 mL each) were collected from the pigs and sent to Lezen Lab in Taipei, Taiwan, for comprehensive analysis. This analysis encompassed parameters including blood urea nitrogen (BUN), creatinine, glucose, total cholesterol, triglycerides, calcium, high-density lipoprotein cholesterol (HDL-C), low-density lipoprotein cholesterol (LDL-C), immunoglobulin G (IgG), albumin, globulin, and complete blood count (CBC).

### Analysis of fecal enzyme activity and odor-related metabolites

Fecal samples (1 g) were homogenized in phosphate-buffered saline (PBS), followed by centrifugation to obtain the supernatant for enzyme and metabolite analyses. Ammonia nitrogen concentrations, indicative of ammonia gas and fecal odor, and protease activity were measured according to the method of Shriver et al. [28]. Protease activity was analyzed by using azocasein as the substrate in potassium phosphate buffer. After incubation, trichloroacetic acid (TCA) was added to stop the reaction, followed by centrifugation and absorbance measurement at 450 nm. Urease activity was evaluated using a commercial Urease Activity Assay Kit (BioVision, Milpitas, CA, USA) as per the manufacturer’s instructions. Fecal odor-related compounds including indole, skatole, and p-cresol were quantified using high-performance liquid chromatography (HPLC) as described by Shriver et al. [28]. Fecal homogenates were extracted with PBS and acetonitrile, centrifuged (13,000 × g, 10 min), and filtered through a 0.22 μm PTFE membrane. Separation was performed on a LiChrospher^®^ 100 RP-18 (5 μm) LiChroCART^®^ 250-4 column (Merck KGaA, Darmstadt, Germany), with a mobile phase consisting of 200 mM ammonium formate (pH 4.5) and acetonitrile (52:48, v/v). The flow rate was 0.5 mL/min, column temperature 30 °C, and sample injection volume 20 µL. Detection was conducted by fluorescence with excitation/emission wavelengths of 270/340 nm. The acetic acid to propionic acid (A: P) ratio was determined using HPLC. Fecal samples were homogenized with 0.1 M sulfuric acid, centrifuged, and filtered (0.22 μm PTFE) before injection. Organic acid separation was carried out on a Rezex™ ROA-Organic Acid H⁺ (8%) column (300 × 4.6 mm, Phenomenex, Torrance, CA, USA), with 0.01 N sulfuric acid as the mobile phase at a flow rate of 0.5 mL/min and a column temperature of 40 °C. Detection was performed by UV absorbance at 210 nm, and concentrations were calculated based on external standard calibration curves.

### Analysis of carcass traits and grading

The analysis encompasses a range of parameters, including carcass percentage (%), slaughtering weight (kg), carcass length (cm), backfat thickness (cm), loin area (cm²), lean percentage (%), bone% (%), pH value, fat percentage (%), and weights of front, middle, and back sections of various parts. The carcass length was measure form the front edge of the first rib to the front of the pubic bone. The backfat thickness was measured by measuring the backfat thickness of the first rib, the last rib and the last lumbar vertebra 5 cm above the left longissimus dorsi muscle, and then finding the average value. The definition of the front, middle, and back section of carcass was from the head to the tail, the cutting points were between the 4th and 5th ribs, and between the 5th and 6th lumbar vertebrae. After cutting the left longissimus dorsi muscle between the tenth and eleventh ribs, the color, marbling score and cross-section of loin area were measured. The left longissimus dorsi muscle from the 5th to 10th rib was sampled for the measurement of meat quality.

Slaughtering weight was measured after bleeding and the removal of internal organs, head, skin, feet, tail, and leaf lard. Carcass percentage was calculated by taking (weight of the carcass / weight of live animal) ×100. Carcass length, backfat thickness, and loin area were measured using established anatomical landmarks and planimetry techniques. Lean and bone percentages were calculated based on specific anatomical components relative to slaughtering weight. pH value was measured using a microcomputer pH meter inserted into the longest back muscle. Fat percentage was determined by analyzing back fat and subcutaneous fat relative to slaughtering weight. The meat quality score was assessed according to established color, firmness, and marbling criteria. General muscle composition was analyzed following AOAC (2005) methods [29], while drip loss, cooking loss, shear force, and color measurements were conducted using standardized procedures.

### Cecal microbiota DNA extraction and 16 S rDNA sequencing

Cecum contents were collected using ESwab™ 480 C swabs with liquid Amies medium (COPAN Diagnostics, Italy), with ~ 1 g of sample transferred into the swab medium and shaken for 30 s. DNA extraction was performed as previously described [30], with modifications. Samples were centrifuged, and the pellets were lysed in DNA extraction buffer using a FastPrep-24™ 5G bead beater (MP Biomedicals). Following PCI (phenol: chloroform: isoamyl alcohol, 25:24:1) extraction and centrifugation, DNA was precipitated with sodium acetate and isopropanol, washed with 70% and 99% ethanol, and resuspended in TE buffer for storage at − 20 °C.

Full-length bacterial 16 S rDNA was amplified using barcoded primers (forward: AGRGTTYGATYMTGGCTCAG; reverse: RGYTACCTTGTTACGACTT), and sequencing was conducted on the PacBio HiFi platform following manufacturer guidelines (Amplification of bacterial full-length 16 S gene with barcoded primers, Part Number 101–599–700 Version 05, PacBiO) [31]. Raw reads were denoised using DADA2 (v1.20) [32], and Amplicon Sequence Variants (ASVs) were assigned using QIIME2 [33] with taxonomic classification via the classify-consensus-vsearch function based on NCBI 16 S, GreenGenes2 (2022.10) [34], and SILVA (v132/138) [35] databases. Microbial community differences were visualized using PLS-DA plots (R packages mixOmics and ggplot2), while LEfSe analysis (bioBakery and microeco) identified discriminative taxa (LDA score > 4).

### Statistical analysis

The data analysis in the present study utilized Statistical Analysis System (SAS) v9.4 software (SAS Institute Inc., SAS Campus Drive, Cary, North Carolina, USA). Results were presented as mean ± standard deviation (mean ± SD). Data that adhered to a normal distribution was analyzed using one-way ANOVA, followed by Tukey’s post hoc test; otherwise, non-parametric analysis employing the Kruskal–Wallis test with Dunn’s post hoc comparison was conducted to assess differences in median values between data groups. Result graphs were generated using Prism v8.0 software (GraphPad Software Inc., San Diego, CA, USA). Given that microbial analysis data were expressed as relative abundance, non-parametric statistical analysis was employed to evaluate differences in gut microbiota between groups. The Kruskal–Wallis test assessed differences in median values between data groups. The relationship between gut microbiota and relevant physiological indicators was statistically evaluated in correlation analysis using the Spearman correlation coefficient. Finally, result data visualizations were generated using Prism 8.00 (GraphPad Software Inc., San Diego, CA, USA).

## Results

### Probiotic supplementation downregulated diarrhea and upregulated growth performance

**Fig. 1:**
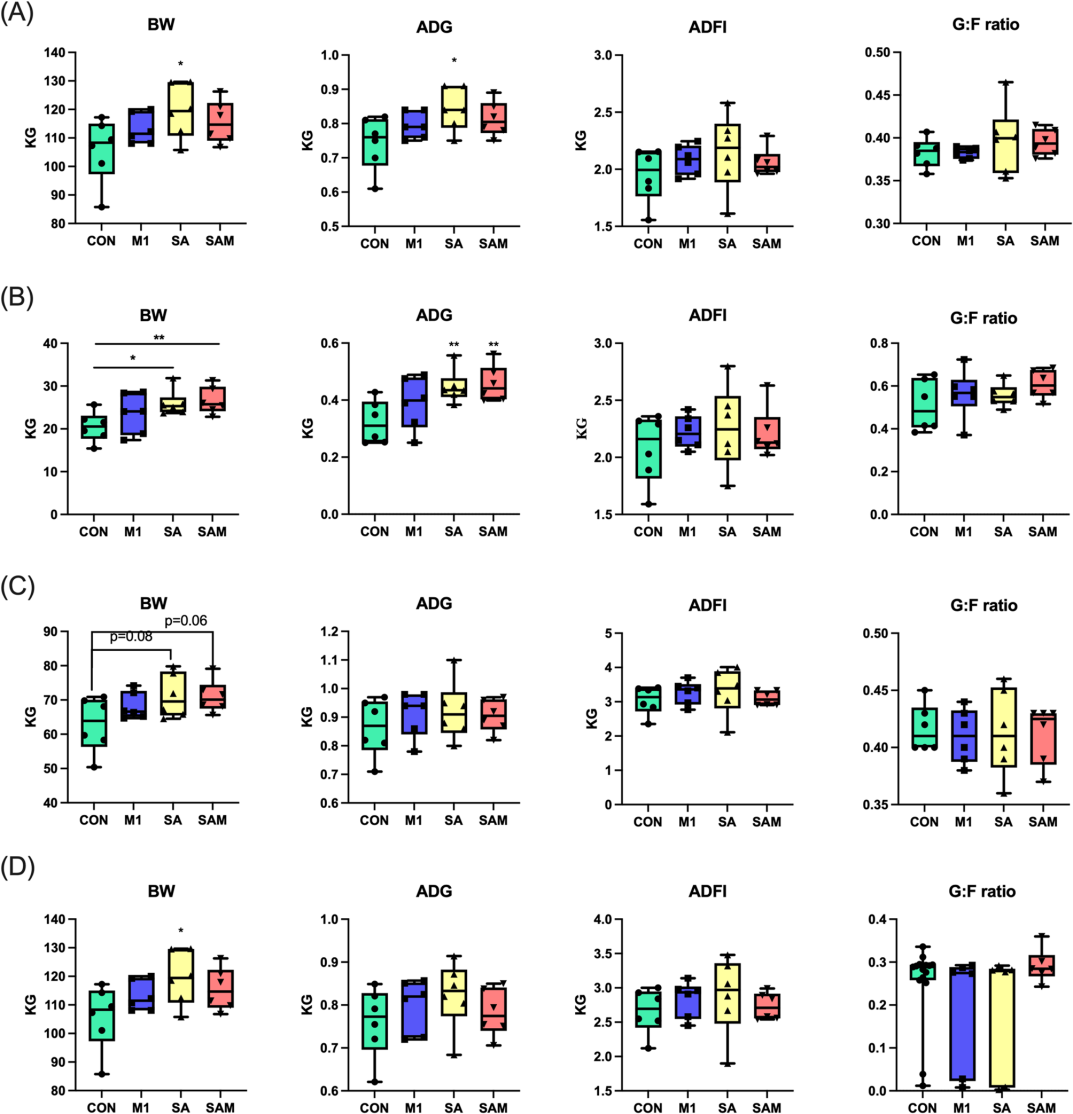
Effects of probiotic supplements on growth performance of (**A**) full term, (**B**) nursery, (**C**) growing, and (**D**) finishing pigs. (continued) *P < 0.05, **P < 0.01. BW: body weight. ADG: average daily weight gain. ADFI: average daily feed intake. G: F ration: gain-to-feed ratio

Initially, we assessed the impact of probiotic supplementation on growth performance. Over the entire study duration (4 to 23 weeks of age), the SA group demonstrated a notable increase of 10.89% and 13.01% in body weight (BW) and average daily gain (ADG), respectively, compared to the Control group (*P* < 0.05). Similarly, the SAM group exhibited a trend towards elevated BW and ADG (Fig. 1A). During the nursery phase (4 to10 weeks of age), both probiotic groups (SA, SAM) displayed significant enhancements in BW and ADG (*P* < 0.05). Specifically, BW surged by 27.02% and 29.09% in the SA and SAM groups, respectively, compared to the Control. Likewise, ADG rose by 38.39% and 41.49% in the SA and SAM groups, respectively (Fig. 1B). Moving into the growing and finishing phases, the SA and SAM groups consistently exhibited higher BW than the Control group (Fig. 1C and D). However, no significant disparities were observed in average daily feed intake (ADFI) and gain-to-feed (G: F) ratio across all study periods.

The daily incidence rate of diarrhea in weaned piglets was closely monitored. After a 4-week period, the Control group exhibited a significantly higher diarrhea score (*P* < 0.05) and diarrhea frequency compared to both the M1 and SAM groups (Table 1). Furthermore, analysis of blood biochemistry before slaughter (Table S1 and Table S2) revealed no notable variances among the groups, suggesting the absence of adverse effects on essential physiological functions attributable to probiotic intake. Throughout the study duration, the pigs consistently maintained their health and well-being.

### Evaluation of the effects of probiotic supplementation on odor-related metabolites in pigs

**Fig. 2:**
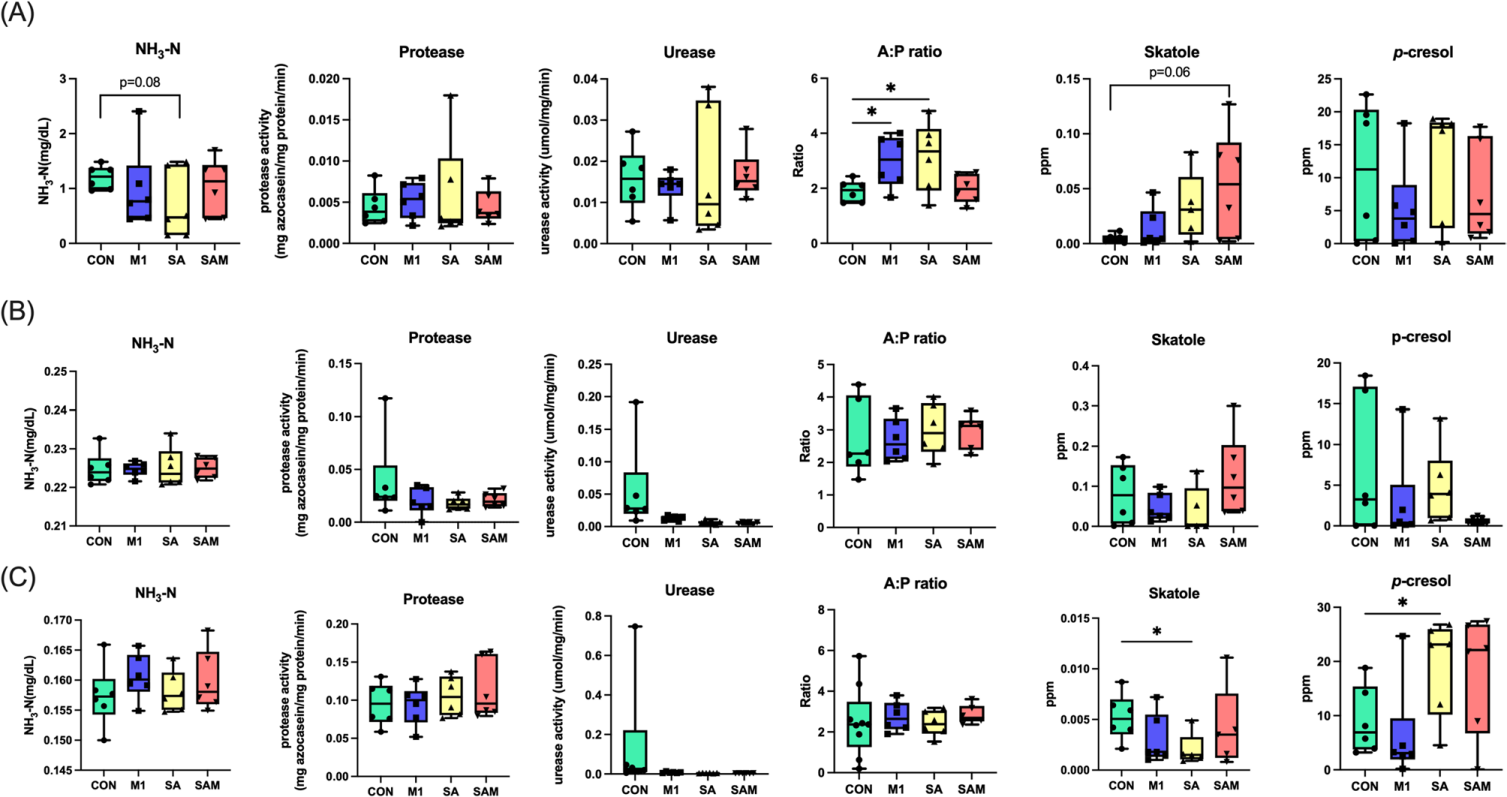
Effects of probiotic supplements on enzyme activity and metabolite odor substances of (**A**) nursery, (**B**) growing, and (**C**) finishing pigs *P < 0.05

To assess the potential impact of probiotic supplementation on mitigating odor in pig feces, we examined fecal parameters including fecal ammonia nitrogen, enzyme activity (protease and urease), and metabolites across various growth stages. During the nursery phase, the M1 and SA groups exhibited a significant increase in the acetate-to-propionate (A: P) ratio compared to the Control group (*P* < 0.05). Additionally, ammonia nitrogen showed a rising trend, while skatole displayed a decreasing trend in the SA group (0.5 < *P* ≤ 0.1) (Fig. 2A). In the finishing phase, skatole levels were significantly reduced, while p-cresol levels were elevated in the SA group (*P* < 0.05) (Fig. 2B). However, there were no significant differences observed in protease and urease levels in fecal samples among the three groups across the various growth stages (Fig. 2C). These findings suggested that the probiotics we used may have a limited effect on modulating odor-related metabolites.

### Probiotic supplementation improves carcass grading and meat quality

**Fig. 3:**
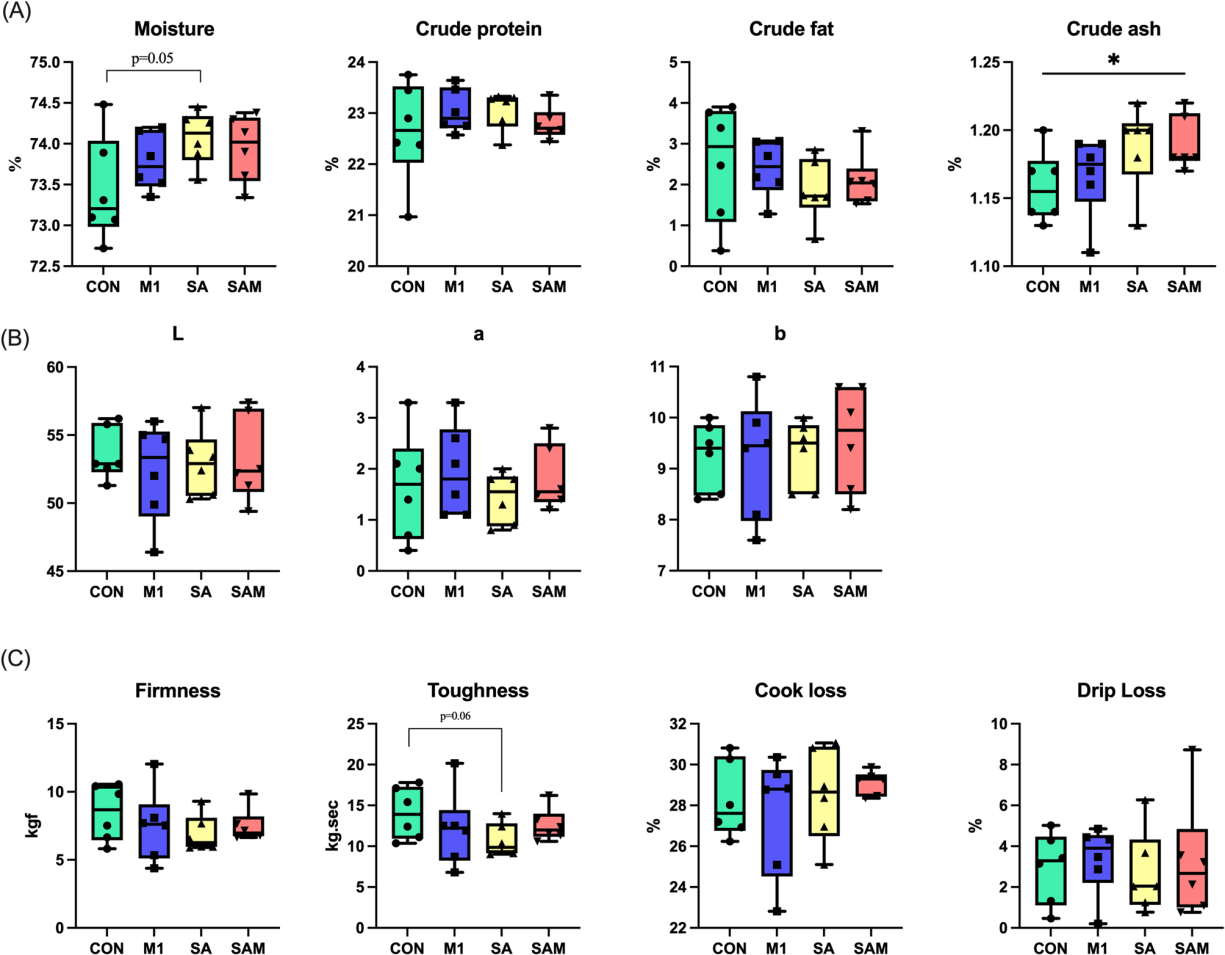
Effects of probiotic supplements on meat quality in (**A**) proximate composition, (**B**) color, (**C**) firmness and hydrated of finishing pigs. *P < 0.05

The impact of probiotic supplementation on carcass and meat quality was thoroughly examined, with results summarized in Table 2. Notably, compared to the Control group, the SA group exhibited a significantly higher carcass percentage (*P* < 0.05), while slaughter weight, pork neck weight, and tenderloin weight were slightly higher, though without statistical significance. Similarly, the SAM group exhibited significantly elevated levels of tenderloin weight compared to the Control group (*P* < 0.05), along with a non-significant trend toward increased weights of the picnic, shoulder, flank, and hand shank portions. These observations suggest a potential additive effect of multi-strain probiotic supplementation on carcass composition, although not all parameters reached statistical significance. Moreover, analysis of meat quality (Fig. 3) revealed that the moisture content in the SA and SAM groups was significantly higher than in the Control group (*P* < 0.05). In contrast, although reductions in pork toughness and firmness were observed in these groups, these differences did not reach statistical significance, indicating only a numerical trend toward improved texture.

**Table 2:** Effects of probiotic supplements on carcass grading in pigs

	CON	M1	SA	SAM	*p*-value
Live weight (kg)	114.96 ± 11.27	119.38 ± 6.57	124.51 ± 9.71	122.75 ± 7.63	0.2974
Slaughtering weight (kg)	96.43 ± 10.41	101.51 ± 6.13	106.55 ± 8.98	103.98 ± 7.29	0.2216
Carcass percentage (%)	83.80 ± 1.57	85.02 ± 0.87	85.54 ± 0.92	84.68 ± 0.89	0.0798
Carcass long (cm)	94.79 ± 3.83	96.17 ± 2.21	97.21 ± 2.87	96.67 ± 1.36	0.3285
Backfat thickness (cm)	1.95 ± 0.45	2.03 ± 0.52	2.01 ± 0.39	1.74 ± 0.52	0.5965
Lean percentage (%)	51.44 ± 2.78	51.09 ± 3.21	51.25 ± 2.35	52.62 ± 1.33	0.4724
Fat percentage (%)	12.31 ± 2.23	12.26 ± 3.52	13.51 ± 3.47	10.56 ± 2.47	0.4765
Bone% (%)	15.83 ± 0.55	15.52 ± 0.80	15.08 ± 0.87	15.84 ± 1.20	0.7159
**pH value**					
Ham (1 h)	6.258 ± 0.151	6.242 ± 0.254	6.308 ± 0.141	6.126 ± 0.223	0.4562
Loin (1 h)	6.336 ± 0.159	6.282 ± 0.272	6.266 ± 0.142	6.361 ± 0.161	0.8035
Ham (24 h)	5.716 ± 0.108	5.803 ± 0.363	5.769 ± 0.081	5.687 ± 0.066	0.7392
Loin (24 h)	5.797 ± 0.088	5.786 ± 0.128	5.798 ± 0.091	5.820 ± 0.122	0.1038
**Front section**					
Shoulder (kg)	5.021 ± 0.740	5.214 ± 0.759	5.463 ± 0.431	5.627 ± 0.349	0.3355
Boston butt (kg)	2.780 ± 0.359	2.847 ± 0.412	3.089 ± 0.311	2.986 ± 0.398	0.4913
Shank (kg)	0.258 ± 0.035	0.257 ± 0.032	0.251 ± 0.032	0.288 ± 0.010	0.1474
Pork neck (kg)	1.016 ± 0.151	1.002 ± 0.230	1.151 ± 0.130	0.972 ± 0.227	0.3943
**Middle section**					
Loin (kg)	3.836 ± 0.737	4.110 ± 0.499	4.335 ± 0.264	4.102 ± 0.537	0.4736
Loin area (cm^2^)	57.84 ± 10.63	58.22 ± 12.83	59.74 ± 5.92	61.07 ± 8.18	0.9358
Belly (kg)	4.056 ± 0.484	4.261 ± 0.624	4.447 ± 0.507	4.564 ± 0.546	0.4110
Tenderloin (kg)	0.545 ± 0.081	0.612 ± 0.092	0.616 ± 0.071	0.641 ± 0.041	0.1715
**Back section**					
Ham (kg)	7.906 ± 1.385	8.190 ± 0.900	8.581 ± 0.665	8.626 ± 0.437	0.4918
Hind shank (kg)	0.469 ± 0.046	0.477 ± 0.062	0.477 ± 0.052	0.519 ± 0.040	0.3261

Data were expressed as mean ± SD, *n* = 6 per group. ANOVA was applied to evaluate the significant difference with all groups

### Probiotic supplementation changed the population of specific bacterial species

**Fig. 4:**
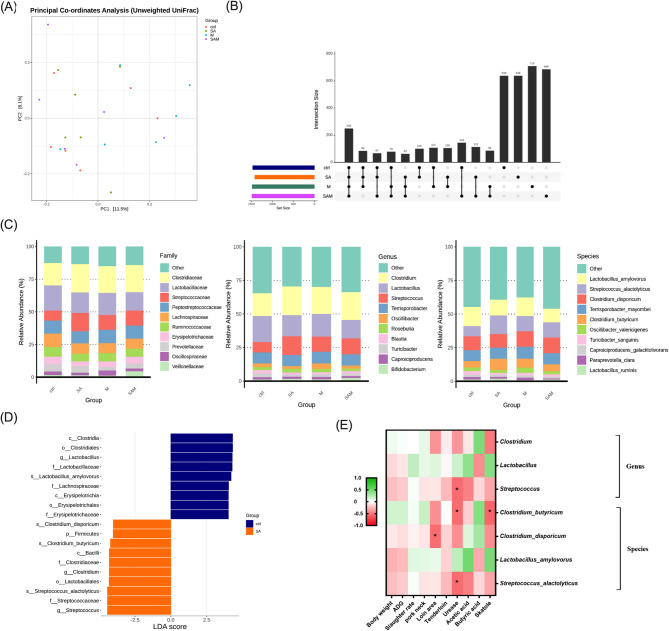
The effect of probiotic supplementation on cecum microbiota composition in pigs. (**A**) Principal Coordinates Analysis (PCoA) and (**B**) UpSet plot of the ASVs of cecum microbiota (**C**) The distribution of 10 predominant bacterial taxa in cecum microbiota at family, genus, and species level. (**D**) Cladogram of cecum microbiota generated from linear discriminant analysis effect size (LEfSe) analysis, illustrating the bacterial differences between the Control and SA groups (LDA score: 4.0). (**E**) Results of the Spearman’s correlation test between phenotypes and the bacterial taxa identified by LEfSe at both the species and genus levels. Ctrl = Control group (basal diet without probiotic supplementation)

After confirming the beneficial effects of probiotic supplementation in pigs, we performed full-length 16 S rRNA gene sequencing of the cecal microbiota to investigate the relationship between probiotic supplementation and gut microbial composition. The sequencing data, including the number of raw, filtered, and denoised reads per sample processed with DADA2, are summarized in Table S4. Alpha diversity metrics, including the Chao1 richness estimator and Shannon diversity index, showed no significant differences among the four groups (*P* > 0.05; Figure S1). Beta diversity was assessed using Principal Coordinates Analysis (PCoA; Fig. 4A) based on Bray-Curtis distances, and group differences were further evaluated using PERMANOVA (Adonis) and ANOSIM. The results demonstrated no significant differences in overall microbial community composition between groups at either the genus level (PERMANOVA: F = 0.073, *P* = 0.89; ANOSIM: *R* = -0.031, *P* = 0.34) or the species level (PERMANOVA: F = 0.087, *P* = 0.82; ANOSIM: *R* = -0.018, *P* = 0.51). These findings indicate that probiotic interventions did not significantly affect the overall beta diversity of the gut microbiota. Additionally, the Upset diagram in Fig. 4B shows that 249 ASVs were shared across all groups, while the Control, M1, SA, and SAM groups harbored 638, 638, 710, and 686 unique ASVs, respectively.

The top 10 dominant taxa at the family level, collectively covering 80% of the total genus, were identified across groups, albeit with varying proportions (Fig. 4C). Clostridiaceae emerged as the most prevalent family (19.99%), succeeded by Lactobacillaceae (16.49%), Streptococcaceae (11.17%), Peptostreptococcaceae (9.84%), Lachnospiraceae (8.42%), Ruminococcaceae (6.38%), Erysipelotrichaceae (4.78%), Prevotellaceae (4.20%), Oscillospiraceae (2.74%), and Veillonellaceae (2.13%). At the genus level, *Clostridium* (19.78%) dominated, followed by *Lactobacillus* (16.47%), *Streptococcus* (11.16%), *Terrisporobacter* (8.44%), and *Oscillibacter* (2.74%).

A total of 196 bacterial species were identified in the gut microbiome. Among them, *Lactobacillus amylovorus* (12.48%), *Streptococcus alactolyticus* (11.04%), and *Clostridium disporicum* (10.92%) were the most dominant across all groups (Fig. 4C). Notably, six species showed significant differences among the four treatment groups (*P* < 0.05), including *Solobacterium moorei*, *Thiobacillus denitrificans*, *Clostridium saccharoperbutylacetonicum*, *Clostridium chartatabidum*, *Rugosibacter aromaticivorans*, and *Blautia obeum* (Table 3).

**Table 3:** The relative abundance (%) of significant different dominant bacterial taxa in 24 cecum samples at genus and species level

Genus	Whole (*n* = 24)		CON (*n* = 6)		M1 (*n* = 6)		SA (*n* = 6)		SAM (*n* = 6)		*p*-value
	Mean	SD	Mean	SD	Mean	SD	Mean	SD	Mean	SD	
*Solobacterium*	0.564	0.235	0.595^AB^	0.218	0.382^B^	0.123	0.494^AB^	0.176	0.784^A^	0.235	0.0409
*Thiobacillus*	0.332	1.024	0.000^B^	0.000	1.217^A^	1.867	0.109^AB^	0.267	0.000^B^	0.000	0.0167
*Rugosibacter*	0.190	0.644	0.000 ^B^	0.000	0.718 ^A^	1.205	0.043^AB^	0.106	0.000 ^B^	0.000	0.0032
*Sharpea*	0.100	0.202	0.000 ^B^	0.000	0.027^AB^	0.041	0.081^AB^	0.085	0.292^A^	0.340	0.0063
*Bacteroides*	0.052	0.093	0.150 ^A^	0.127	0.011 ^AB^	0.018	0.048^AB^	0.080	0.000 ^B^	0.000	0.0153
**Species**											
*Solobacterium_moorei*	0.564	0.235	0.595^AB^	0.218	0.382^B^	0.123	0.494^AB^	0.176	0.784^A^	0.235	0.0409
*Thiobacillus_denitrificans*	0.318	0.983	0.000 ^B^	0.000	1.165 ^B^	1.793	0.109^AB^	0.267	0.000^B^	0.000	0.0167
*Clostridium_saccharoperbutylacetonicum*	0.213	0.221	0.059 ^B^	0.091	0.127 ^AB^	0.104	0.253^AB^	0.127	0.415 ^A^	0.317	0.0167
*Clostridium_chartatabidum*	0.201	0.257	0.157	0.367	0.057	0.089	0.285	0.256	0.306	0.216	0.0406
*Rugosibacter_aromaticivorans*	0.190	0.644	0.000 ^B^	0.000	0.718^A^	1.205	0.043^AB^	0.106	0.000 ^B^	0.000	0.0032
*Blautia_obeum*	0.189	0.156	0.329 ^A^	0.179	0.082 ^B^	0.113	0.198^AB^	0.105	0.147 ^AB^	0.128	0.0253

Data were expressed as mean ± SD. The Kruskal-Wallis test was applied to evaluate the significant difference with all groups. ^A, B^ Means within row with different superscripts differ by Dunn’s test (*P* < 0.05)

### Identification of the differential abundance of gut bacteria

The results from evaluations of growth performance, carcass grading, and meat quality highlighted the superior efficacy of SA supplementation compared to other groups. Consequently, the taxa associated with the SA and Control groups were subjected to analysis using the LEfSe algorithm, with LDA > 4.0 employed to identify the differential abundance. This analysis revealed 19 influential taxonomic clades, encompassing three genera and four species (Fig. 4D). In the Control group, the differential abundance of taxa included one genus (*Lactobacillus*) and one species (*Lactobacillus_amylovorus*). In contrast, the SA group exhibited two genera (*Clostridium*, *Streptococcus*) and three species (*Clostridium_disporicum*, *Clostridium_butyricum* and *Streptococcus alactolyticus*) as associated taxa. These findings elucidate that the specific microbial taxa are associated with probiotic supplementation, offering valuable insights into their potential impact on gut microbiota composition.

Further analysis using Spearman’s correlation coefficient was conducted to explore the associations between gut microbiota and various phenotypic traits. As illustrated in Fig. 4E, the heatmap unveiled noteworthy negative correlations among pork neck, urease, and skatole (*P* < 0.05). Specifically, *Clostridium_butyricum* demonstrated a significant negative correlation with skatole and urease (*P* < 0.05), whereas *Clostridium_disporicum* displayed a significant negative correlation solely with urease (*P* < 0.05).

## Discussion

Our study conducted a comprehensive analysis of probiotic effects throughout all stages of pig rearing, spanning from piglets to finishing pigs. We assessed various parameters including health indicators, growth performance, fecal odor emission, and carcass quality. By integrating analyses of the gut microbiome and observed parameters, we gained deeper insights into the mechanisms underlying the beneficial effects of probiotics, elucidating microbiome composition and host metabolites contributing to these effects.

Initially, we observed a significant enhancement in pig growth, particularly noteworthy during the crucial nursery period, facilitated by probiotic intervention. This increase in body weight had cascading effects on both slaughtering weight and percentage, key indicators of revenue generation. In contrast to our findings, prior studies [36, 37] suggested that probiotic supplementation has limited impact on the growth performance of growing pigs [38]. Despite the relatively stable intestinal microbiota, heightened immune status, and improved digestion efficiency of growing and finishing pigs compared to weaned piglets [39], early and prolonged use of probiotics still contributes to growth performance in the later stages of the growth phase.

Additionally, we observed that the SA group demonstrated superior growth performance compared to the M1 group, contrary to our expectations. In our previous research, administering *L. kefiranofaciens* M1 resulted in a significant increase in body weight in C57BL/6J male mice fed a high-fat diet [19]. The previous results showed that the efficacy of *L. kefiranofaciens* M1 was enhanced in the presence of a high-fat diet, as it may modulate lipid metabolism pathways more effectively under such conditions. In contrast, the standard swine diet used in the present study may not provide the same metabolic triggers. The difference may be explained by host-specific responses and different diet composition. These results indicated the importance of strain–host–diet interactions and underscore the need to tailor probiotic strategies to specific nutritional contexts and animal models. Besides growth performance, we also observed a pronounced reduction in diarrhea incidence during nursery period after one month of probiotic supplementation in two probiotic groups containing *L. kefiranofaciens*. The observed decrease in diarrhea incidence during the nursery phase, a period often associated with heightened susceptibility to diseases [40], may be attributed to the functional properties of the specific strain, *L. kefiranofaciens* M1. This strain has demonstrated gut barrier protection [21], anti-colitis properties [18], and immunoregulatory effects [41] both in vitro and in vivo.

In terms of odor reduction, during the finishing period, the experimental groups supplemented with SA notably decreased the concentration of fecal odor compounds, particularly skatole, which is a primary contributor to the odor in swine house air [42]. *Bacillus* species have demonstrated the ability to degrade volatile sulfur compounds such as hydrogen sulfide [43, 44], leading to reduced ammonia and hydrogen sulfide emissions in various studies [45, 46]. Additionally, *Bacillus* supplementation may enhance the utilization of sulfur-containing amino acids in the host, thereby decreasing skatole production. Although the use of SA shows promises in reducing certain odor indicators, further evidence is needed to substantiate its effectiveness in odor reduction.

Through post-slaughter analysis of pig carcass characteristics and meat quality in this study, the probiotic supplement SA group showed a significant increase in slaughter yield, improved water-holding capacity, and better meat color. This aligns with findings from previous study [47], indicating that probiotics can positively impact carcass weight and grading, corroborating our results. Probiotics may influence muscle composition and structure, thus impacting water retention in meat, which in turn can elevate meat quality parameters such as tenderness and juiciness [48]. The color of meat, influenced by factors like myoglobin content and oxidation, can also be modulated by probiotics. Recent research highlights the role of probiotics in mitigating myoglobin oxidation, thereby contributing to enhanced meat coloration [49]. However, existing literature [50, 51] suggests that the addition of probiotics to pig feed during the finishing period typically has minimal effects on carcass characteristics. Such variability underscores the multifaceted nature of factors affecting carcass traits, including age, breed, nutrition, feeding practices, and gender [52], further research is warranted to elucidate the primary factors affecting carcasses characteristics.

It is important to recognize that the specific impacts of probiotics on beneficial traits can be influenced by various factors including the strains utilized, dosage, growth stage, duration of administration, and the overall health status and circumstances of the pigs. Based on research findings, a regimen involving the concurrent administration of three probiotic strains (*L. kefiranofaciens* M1, *B. subtilis* S14, and *B. amyloliquefaciens* S20) during the nursery period, along with the inclusion of *B. subtilis* S14 and *B. amyloliquefaciens* during the growing and finishing phases, is recommended. Although we observed that the SAM group showed no significant improvements in growth performance. This may be attributed to inter-strain competition, functional overlap, or reduced colonization efficiency when multiple strains are combined. Similar findings have been reported in other multi-strain probiotic studies [53, 54].

Maintaining a balanced and healthy gut microbiome is essential for optimal nutrient absorption and digestive well-being. The cecum microbiome analysis with UpSet plots underscored notable alterations across treatment groups in pigs. When comparing the dominant bacterial taxa among the four treatment groups, significant differences were observed in the abundance of *Rugosibacter_aromaticivorans*. *Rugosibacter_aromaticivorans* has been linked to the degradation of polycyclic aromatic hydrocarbons (PAHs) [55–59]. Exposure to PAHs, whether short-term or long-term, is associated with potential harm to bodily fluids, skin, and the immune system. Additionally, *Clostridium_saccharoperbutylacetonicum*, recognized as a gram-positive, spore-forming anaerobic bacterium, also exhibited significant differences among the treatment groups. This species, known for its capacity to produce high levels of acetone and butyric acid, plays a role as a cellulose degrader and has been linked to positive effects on nutrient digestion and gut health. The observed upregulation of *Rugosibacter_aromaticivorans* and *Clostridium_saccharoperbutylacetonicum* in the probiotic intervention groups implies potential benefits for their overall health and well-being.

The analysis further underscored the notable influence of the SA group on the microbial community within the pig cecum. Specifically, genera *Clostridium* and *Streptococcus*, alongside *Clostridium butyricum*, *Clostridium_disporicum*, and *Streptococcus_alactolyticus* within these genera, emerged as biomarkers within the SA group. *Clostridium butyricum*, renowned for its production of short-chain fatty acids (SCFAs) such as butyrate and acetate, plays a pivotal role. SCFAs, synthesized by microbial organisms in the colon, wield a diverse array of crucial effects on host health [60, 61]. *Streptococcus alactolyticus*, identified as the predominant culturable lactic acid bacteria species in both dog feces and intestines [62], has also been documented in pigeon intestines, pig intestines, and chicken feces, albeit as a minor component of the microbiota [63–65]. Studies have shown its non-toxic nature and its ability to enhance the health of broilers infected by *E. coli* O78, by protecting the intestinal villi structure, maintaining intestinal immune balance, and exerting antioxidative effects [66]. The identification of biomarkers within the SA group offers a potential link to unraveling the intricate role of gut microbiota and their myriad functions.

Reducing odor and skatole in pig growth is a multifaceted process influenced by various metabolic pathways, wherein probiotics may exert significant effects. Probiotics, such as SA, enhance carbohydrate metabolism by upregulating *Clostridium_butyricum* and *Streptococcus_alactolyticus*, fostering a balanced gut microbiota and promoting the production of beneficial short-chain fatty acids. Additionally, the activation of styrene degradation mechanisms can help mitigate the presence of styrene, a volatile organic compound with a distinct odor. Styrene degradation by microbes involves specific enzymatic pathways [67, 68]. Although the direct relationship between probiotics and styrene degradation is not fully established, administering a mixture of *B. subtilis* S14 and *B. amyloliquefaciens* S20 could promote a healthy gut microbiota and indirectly contribute to overall metabolic balance, potentially influencing the fate of certain environmental compounds.

## Conclusion

Here, a thorough examination of probiotic effects across all stages of pig rearing, integrating results pertaining to health indicators, growth performance, fecal odor emission, and carcass quality, underscores the importance of long-term probiotic supplementation and the necessity of adjusting probiotic strains according to varying needs during different stages. We also uncovered intervention with *L*. *kefiranofaciens* M1, *B. subtilis* S14 and *B. amyloliquefaciens* S20 modulated microbial composition, contributing to the downregulation of fecal odor emission and enhancement of growth performance. These findings pave the way for further research into the complex role of gut microbiota in host health and homeostasis.

## Electronic supplementary material

Below is the link to the electronic supplementary material.

Supplementary Material 1

## Data Availability

The sequencing data supporting the finding of this study have been deposited in the NCBI with the accession code PRJNA1120206.
