# Supplementary material for: Probiotic supplementation regulated swine growth performance, fecal odor reduction and carcass characteristics by modulating intestinal microbiome
Source: Anim Microbiome. 2025 Jul 16;7:74. doi: 10.1186/s42523-025-00441-9 (PMC12269218; doi:10.1186/s42523-025-00441-9)
Supplement: Supplementary file 1 — Supplementary Material 1 [file 42523_2025_441_MOESM1_ESM.docx]

**Probiotic Supplementation Regulated Swine Growth Performance, Fecal Odor Reduction and Carcass Characteristics by Modulating Intestinal Microbiome**

Yung-Tsung Chen^a#^, Yu-Ting Sun^b#^, Herng-Fu Lee^c^, Yu-Chun Lin^d^, Ming-Ju Chen^b*^

^a^ Department of Food Science, National Taiwan Ocean University, Taiwan

^b^ Department of Animal Science and Technology, National Taiwan University, Taiwan

^c^ Taiwan Livestock Research Institute, Ministry of Agriculture, Taiwan

^d^ Fisheries Research Institute, Ministry of Agriculture, Taiwan

# Yung-Tsung Chen and Yu-Ting Sun contributed equally to this work.

* Corresponding author.

Email address: cmj@ntu.edu.tw (M.-J. Chen)

**Supplementary Data**

**Table S1 Ingredient and analyzed values of the diets**

| **Ingredient (kg)** | **Nursery Phase 1** | **Nursery Phase 2** | **Growth Phase** | **Finishing Phase** |
| --- | --- | --- | --- | --- |
| Corn | 59.37 | 64.20 | 69.51 | 78.00 |
| Soybean Meal | 19.79 | 22.67 | 24.74 | 19.00 |
| Fish Meal | 8.00 | 5.00 | - | - |
| Skim Milk Powder | 6.00 | 2.00 | - | - |
| Whey Powder | 2.00 | 2.00 | - | - |
| Salad Oil | 2.00 | 1.20 | 1.20 | 0.20 |
| Molasses | - | - | 1.50 | - |
| Salt | 0.50 | 0.50 | 0.50 | 0.50 |
| Choline Chloride (50%) | 0.10 | 0.10 | 0.10 | 0.05 |
| Dicalcium Phosphate | 0.60 | 0.70 | 1.30 | 1.20 |
| Calcium Carbonate | 0.60 | 0.60 | 0.70 | 0.60 |
| Lysine | 0.42 | 0.42 | 0.20 | 0.20 |
| DL-Methionine | 0.17 | 0.19 | - | - |
| Hydroxybutyric Acid | 0.20 | 0.17 | - | - |
| Mineral Premix ^a^ | 0.15 | 0.15 | 0.15 | 0.15 |
| Vitamin Premix ^b^ | 0.10 | 0.10 | 0.10 | 0.10 |
| Total | 100.00 | 100.00 | 100.00 | 100.00 |
| **Analyzed Values** |  |  |  |  |
| Crude Protein (%) | 20.76 | 19.45 | 15.64 | 13.4 |
| Calcium (%) | 0.94 | 0.87 | 1 | 0.71 |
| Total Phosphorus (%) | 0.60 | 0.57 | 0.51 | 0.47 |
| Metabolizable Energy (kcal/kg) | 4,040 | 3,956 | 3,659 | 3,843 |
| Lysine (%) | 1.56 | 1.53 | 0.87 | 0.82 |
| Sulfur-Containing Amino Acids (%) | 0.72 | 0.73 | 0.43 | 0.40 |
| Hydroxybutyric Acid (%) | 0.96 | 0.92 | 0.57 | 0.52 |

^a^ Vitamin premix provided per kilogram of diet: Vitamin A, 6,000 IU; Vitamin D_3_, 400 IU; Vitamin E, 40 IU; Vitamin K, 2 mg; Vitamin B_1_, 2 mg; Vitamin B_2_, 6 mg; Vitamin B_6_, 3 mg; Vitamin B_12_, 0.03 mg; Niacin, 30 mg; Pantothenic acid, 30 mg; Folic acid, 0.6 mg; and Biotin, 0.2 mg.

^b^ Mineral premix provided per kilogram of diet: Fe, 80 ppm; Cu, 5 ppm; Mn, 6 ppm; Zn, 45 ppm; I, 0.2 ppm; Se, 0.1 ppm; and Co, 0.35 ppm.

**Table S2. Blood biochemical analysis in each group**

| Blood biochemical | CON | M1 | SA | SAM | p-value |
| --- | --- | --- | --- | --- | --- |
| Total cholesterol (mg/dL) | 94.33 ± 14.32 | 84.50 ± 6.16 | 92.17 ± 7.83 | 95.17 ± 9.75 | 0.2662 |
| Triglyceride (mg/dL) | 35.00 ± 14.97 | 38.67 ± 7.79 | 39.83 ± 12.25 | 32.83 ± 7.96 | 0.8856 |
| Albumin (g/dL) | 3.82 ± 0.11 | 3.84 ± 0.21 | 3.89 ± 0.16 | 3.87 ± 0.23 | 0.9222 |
| Globulin (g/dL) | 2.08 ± 0.21 | 1.95 ± 0.26 | 1.97 ± 0.22 | 2.07 ± 0.20 | 0.7273 |
| BUN (mg/dL) | 9.70 ± 2.54 | 9.98 ± 4.23 | 8.95 ± 2.31 | 8.30 ± 2.96 | 0.9471 |
| Creatinine (mg/dL) | 1.51 ± 0.18 | 1.41 ± 0.10 | 1.45 ± 0.09 | 1.43 ± 0.10 | 0.4519 |
| Glucose AC (mg/dL) | 94.50 ± 4.04 | 99.83 ± 6.52 | 93.50 ± 5.82 | 101.00 ± 7.77 | 0.1405 |
| HDL-C (mg/dL) | 42.17 ± 8.71 | 39.03 ± 3.53 | 42.18 ± 4.53 | 43.60 ± 6.13 | 0.5915 |
| LDL-C (mg/dL) | 48.83 ± 6.82 | 42.00 ± 5.06 | 46.33 ± 5.50 | 48.83 ± 4.54 | 0.1267 |
| Calcium (mg/dL) | 10.817 ± 0.366 | 10.667 ± 0.216 | 10.50 ± 0.38 | 10.52 ± 0.54 | 0.3791 |
| IgG (mg/dL) | 476.50 ± 60.434 | 502.67 ± 87.09 | 468.33 ± 67.53 | 497.00 ± 31.81 | 0.8076 |

Data were expressed as mean ± SD. ANOVA was applied to evaluate the significant difference with all groups.

BUN: Blood Urea Nitrogen, Glucose AC: Glucose Ante Cibum, HDL-C: High-Density Lipoprotein Cholesterol, LDL-C :Low-Density Lipoprotein Cholesterol, IgG: Immunoglobulin G.

**Table S3. Blood biochemical complete blood count analysis in each group**

| Complete blood count (CBC) | CON | M1 | SA | SAM | p-value |
| --- | --- | --- | --- | --- | --- |
| WBC (10^6^/µL) | 12.233 ± 2.360 | 11.383 ± 0.408 | 11.967 ± 0.859 | 11.300 ± 1.002 | 0.9801 |
| Neutrophils (%) | 22.550 ± 2.792 | 29.950 ± 1.691 | 27.933 ± 1.313 | 25.767 ± 1.176 | 0.1892 |
| Lymphocytes (%) | 73.083 ± 2.686 | 63.917 ± 2.496 | 68.517 ± 1.478 | 70.633 ± 1.505 | 0.1583 |
| Monocytes (%) | 0.833 ± 0.197 | 0.933 ± 0.248 | 0.883 ± 0.113 | 0.917 ± 0.172 | 0.9933 |
| NLR | 0.323 ± 0.043 | 0.485 ± 0.048 | 0.413 ± 0.028 | 0.370 ± 0.025 | 0.1811 |
| MLR | 0.012 ± 0.003 | 0.014 ± 0.003 | 0.013 ± 0.002 | 0.013 ± 0.003 | 0.7825 |
| Eosinophils (%) | 3.533 ± 0.233 | 3.167 ± 0.756 | 2.667 ± 0.323 | 2.683 ± 0.376 | 0.7297 |
| Basophils (%) | 0.000 ± 0.000 | 2.033 ± 1.502 | 0.000 ± 0.000 | 0.000 ± 0.000 | 0.4133 |
| RBC (10^3^/µL) | 7.323 ± 0.081 | 6.930 ± 0.103 | 7.160 ± 0.150 | 7.085 ± 0.156 | 0.4537 |
| Hemoglobin (g/dL) | 13.517 ± 0.110 | 12.800 ± 0.250 | 13.250 ± 0.237 | 13.000 ± 0.252 | 0.3791 |
| Hematocrit (%) | 41.000 ± 0.508 | 38.833 ± 0.836 | 39.733 ± 0.630 | 39.583 ± 0.846 | 0.4814 |
| M.C.V (fl) | 56.017 ± 0.280 | 55.967 ± 0.523 | 55.567 ± 0.538 | 55.883 ± 0.415 | 0.9535 |
| M.C.H (pg) | 18.450 ± 0.100 | 18.500 ± 0.146 | 18.567 ± 0.221 | 18.350 ± 0.129 | 0.9039 |
| M.C.H.C (g/dL) | 32.950 ± 0.187 | 33.050 ± 0.173 | 33.433 ± 0.134 | 32.817 ± 0.149 | 0.2566 |
| Platelet count (10^3^/µL) | 323.500 ± 19.250 | 335.500 ± 14.539 | 246.000 ± 27.916 | 276.333 ± 39.284 | 0.3012 |

Data were expressed as mean ± SD. ANOVA was applied to evaluate the significant difference with all groups.

WBC: White Blood Cell count, NLR: Neutrophil-to-Lymphocyte Ratio, MLR: Monocyte-to-Lymphocyte Ratio, RBC: Red Blood Cell count, M.C.V: Mean Corpuscular Volume, M.C.H: Mean Corpuscular Hemoglobin, M.C.H.C: Mean Corpuscular Hemoglobin Concentration

**Table S4. The number of raw reads, as well as the filtered and denoised reads per sample processed using DADA2.**

| Sample | Raw HiFi-Read | Filtered Read | Denoised Read | Non-chimera Read |
| --- | --- | --- | --- | --- |
| CON.1 | 13264 | 11732 | 10803 | 9659 |
| CON.2 | 12893 | 11478 | 10494 | 9368 |
| CON.3 | 14893 | 13262 | 12580 | 11154 |
| CON.4 | 13909 | 12288 | 11572 | 10478 |
| CON.5 | 14362 | 12884 | 11879 | 10674 |
| CON.6 | 14214 | 12730 | 12147 | 10954 |
| Total | 83535 | 74374 | 69475 | 62287 |
| M1.1 | 14280 | 12691 | 12010 | 10962 |
| M1.2 | 14822 | 13105 | 12493 | 11474 |
| M1.3 | 13472 | 11969 | 11454 | 9934 |
| M1.4 | 15350 | 13722 | 12510 | 11443 |
| M1.5 | 15756 | 14078 | 13452 | 12312 |
| M1.6 | 14899 | 13274 | 12666 | 11456 |
| Total | 88579 | 78839 | 74585 | 67581 |
| SA.1 | 15815 | 14055 | 13134 | 11950 |
| SA.2 | 14851 | 13216 | 12088 | 10726 |
| SA.3 | 13881 | 12201 | 11577 | 10448 |
| SA.4 | 14307 | 12747 | 12180 | 11065 |
| SA.5 | 15301 | 13598 | 12952 | 11467 |
| SA.6 | 14036 | 12463 | 11810 | 10620 |
| Total | 88191 | 78280 | 73741 | 66276 |
| SAM.1 | 14176 | 12547 | 11856 | 10640 |
| SAM.2 | 14798 | 13099 | 12514 | 11304 |
| SAM.3 | 14907 | 13312 | 11909 | 10784 |
| SAM.4 | 16133 | 14386 | 13394 | 11974 |
| SAM.5 | 15065 | 13579 | 12888 | 11543 |
| SAM.6 | 14511 | 13009 | 12056 | 10914 |
| Total | 89590 | 79932 | 74617 | 67159 |

**Figures**

1. **(B)**


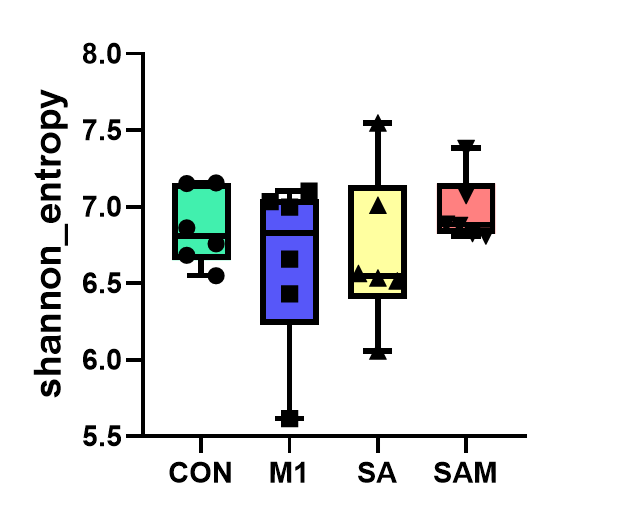


**Figure S1.** (A) Bacterial community richness index estimated by the richness value and (B) community diversity index estimated by estimated by Shannon index in each group.
